# Phytoplankton communities in temporary ponds under different climate scenarios

**DOI:** 10.1038/s41598-021-97516-9

**Published:** 2021-09-09

**Authors:** Sofia Celewicz, Bartłomiej Gołdyn

**Affiliations:** 1 Department of Botany, Faculty of Agronomy, Horticulture and Bioengineering, Poznań University of Life Sciences, Wojska Polskiego 71 C, 60-625 Poznan, Poland; 2 Department of General Zoology, Faculty of Biology, Adam Mickiewicz University in Poznań, 61-614 Poznan, Poland

**Keywords:** Ecology, Plant sciences, Climate sciences, Ecology, Environmental sciences

## Abstract

Temporary water bodies, especially vernal pools, are the most sensitive aquatic environments to climate change yet the least studied. Their functioning largely depends on their phytoplankton community structure. This study aimed to determine how temperature and photoperiod length (by simulating inundation in different parts of the year under five climate scenarios) affect the succession and structure of phytoplankton communities soon after inundation. Photoperiod was the most important factor affecting phytoplankton species richness, total abundance and the abundance of taxonomic groups in the course of succession. A long photoperiod (16 h) and a moderate temperature (16 °C) in vernal pool microcosms (late spring inundation after a warm snowless winter) were the most favourable conditions for phytoplankton growth (especially for the main taxonomic groups: chlorophytes and cryptophytes) and species richness. With short photoperiods (inundation in winter) and low temperatures, the communities transformed towards diatoms, euglenoids and cyanobacteria. In line with our predictions, a high temperature (25 °C) favoured a decline in phytoplankton species diversity. Our study shows that climate change will result in seasonal shifts in species abundance or even in their disappearance and, finally, in potential strong changes in the biodiversity and food webs of aquatic ecosystems in the future.

## Introduction

Climate change caused by global warming has a large impact on aquatic ecosystems, which is particularly relevant in the case of biotic interactions, such as boundary shifts, behavioural and physiological adaptations, or changes in phenology and community structure^1–3^. The specific effects of climate change in aquatic ecosystems (e.g., increase in solar radiation or rainfall and changes in wind speed) will vary among regions and water body types^2^. While global warming has a deleterious impact on freshwater environments^4^, knowledge on its effects on the quantitative and qualitative changes in aquatic communities is not equal for the different types of ecosystems^5^. Numerous studies on climate change impacts have primarily focused on larger and permanent water bodies, such as lakes, sea, and oceans^2,6–9^. There is still a lack of knowledge, however, on the effects of global warming on the functioning of small water bodies (up to one hectare), especially temporary bodies, which are the most sensitive to climate change among aquatic environments^10–12^.

A major feature of temporary water bodies is their cyclic nature, with recurring wet and dry phases. In some cases, this cycle is very regular: under normal conditions, vernal pools fill with water every late winter and become desiccated with the onset of summer. However, it is not clear what will happen if this natural cycle shifts as a result of climate change.

Temporary water bodies are an extremely valuable yet poorly studied type of surface water^13^. As a type of small water body, however, temporary water bodies are perfect sites for a broad range of ecological research and the monitoring of global environmental change. They play vital roles in the human-transformed landscape, constituting significant biodiversity hotspots and taking part in flood control, groundwater recharge, the recycling of nutrients, and toxicant removal^14–17^. In the era of water deficits caused by global climatic changes, their role in stabilising the local groundwater balance is especially important^16^.

Among all types of temporary waters, snow-fed vernal pools seem to be one of the most sensitive to climate change^12^. They are usually shallow, small, and ephemeral aquatic ecosystems with a dry period recurring in late spring every year and lasting until the next year’s snow thaws^13^. Due to their size and drying cycle, they respond rapidly to environmental changes and are characterised by a great fluctuation in abiotic factors^18^. Moreover, the functioning of such ecosystems to a large degree depends on the structure and function of primary producer communities, with phytoplankton being the most important ecological group. Thus, any changes at the base of the aquatic food web are translated into the structure of the whole ecosystem.

After each dry period, phytoplankton communities are formed again, mostly through secondary succession from resting cells preserved in the bottom sediments. The process of recolonisation after the inundation of ponds is crucial for the future structure of communities, but the factors affecting the course of recolonisation and the shaping of algal communities at the beginning of the hydroperiod are still poorly known. Phytoplankton react quickly to changing environmental conditions^19^ due to their short life cycles; thus, phytoplankton serve as a useful model group for research on the influence of environmental variables on the course of such succession. Furthermore, the phytoplankton in temporary ponds are dominated by fast-growing, single-celled r-strategists and opportunists, which are adapted to unstable conditions in rapidly changing environments^13,20,21^. Their structure is subsequently altered by the response of particular species to biotic factors, e.g., macrophytes^22,23^ and filtrators^20^, as well as to abiotic factors, such as temperature, light, pH, and nutrients^23,24^.

Temperature is one of the most important climate-related abiotic factors, and it can strongly influence a phytoplankton community at the onset of a hydroperiod^21,25,26^. Global warming is known to cause changes in phytoplankton community dynamics^27–29^ and species composition and abundance^30,31^, favouring those species that are best adapted to changing conditions. A number of studies (none were conducted on temporary waters) have generally indicated decreasing plankton diversity and increasing picophytoplankton abundance and cyanobacteria blooms as the most evident effects of global warming in water ecosystems^21,26,31–33^.

Another climatic factor determining the functioning of phytoplankton communities is light conditions, on which the photosynthesis of algae largely depends. The light climate—especially in temperate areas at higher geographic latitudes—primarily depends on the length of the day (photoperiod). As such, the light climate is highly related to seasonality, which in turn is strictly dependent on temperature in the case of vernal pools that fill with water from thawing snow. These relations make it difficult to separate the influence of photoperiod length from the influence of other climatic conditions (especially temperature). The species specificity of microalgal light and temperature requirements is not well recognised. Some studies have addressed the effects of temperature or photoperiods under controlled conditions, focusing on select taxonomic groups only, e.g., diatoms, cyanobacteria and chlorophytes. Most of the studies concentrated on particular species and their responses to different temperatures^34^ (e.g., *Spirulina platensis*), photoperiods^35–37^ (e.g., *Alexandrium* sp., *Nannochloropsis* sp., *Tetraselmis* sp., *Thalassiosira* sp.), or both temperature and photoperiod^38,39^ (e.g., *Cryptomonas* sp. and *Dinobryon* sp., *Stephanodiscus minutulus* and *Nitzschia acicularis*). There is a lack of knowledge about the influence of these two factors on whole phytoplankton communities in terms of time, especially for temporary water bodies, and the temporal factor is particularly important considering ongoing climate change.

Our objective was to determine to what degree and in what way photoperiod length and temperature (as a reflection of different climate scenarios) affect the process of secondary succession of algae and the subsequent structure of phytoplankton communities at the onset of the hydroperiod. Hence, following the predictions based on climate change scenarios, we assumed that the shape of the vernal pool phytoplankton community would significantly differ if the start of the water phase was shifted over time (resulting in inundation under atypical day length conditions) or accompanied by altered temperatures.

We conducted experiments under controlled laboratory conditions using a microcosm array, testing for the influence of temperature and photoperiod length on the phytoplankton communities. Our general hypothesis was that these two factors would significantly influence the phytoplankton community structure at the initial stage of succession, and this alteration would translate into the shape of the communities later in the season. The treatments involved particular combinations of three temperatures (4 °C, 16 °C, and 25 °C) and three photoperiod regimes (0 h, 8 h, and 16 h, supplemented by treatments without a period of darkness) as an equivalent to different climate scenarios (for details see “Methods” section), with photoperiod length reflecting the day length when inundation shifts towards winter or late spring/early summer. Our detailed hypotheses were as follows: (1) particular phytoplankton species and taxonomic groups would initiate succession depending on different combinations of temperatures and photoperiod lengths (reflecting particular climate scenarios) so that the succession sequence would differ between the climate scenarios; (2) regardless of the time factor, particular algal groups and species would respond differently to each photoperiod and temperature combination (to different climate scenarios); (3) cyanobacteria would dominate with higher temperatures and longer photoperiods; and (4) the vernal pool phytoplankton diversity would decrease with increasing temperature.

## Results

### Taxonomic richness in particular experimental treatments

In total, 198 phytoplankton taxa from 8 taxonomic groups were identified. The most taxon-rich groups were chlorophytes (80 taxa), euglenoids (43 taxa) and diatoms (34 taxa). Less numerous taxa were cyanobacteria, cryptophytes, dinoflagellates, xanthophytes and chrysophytes (21, 12, 4, 3, and 1 taxa, respectively); for details, see the data in S1.

**Figure 1:**
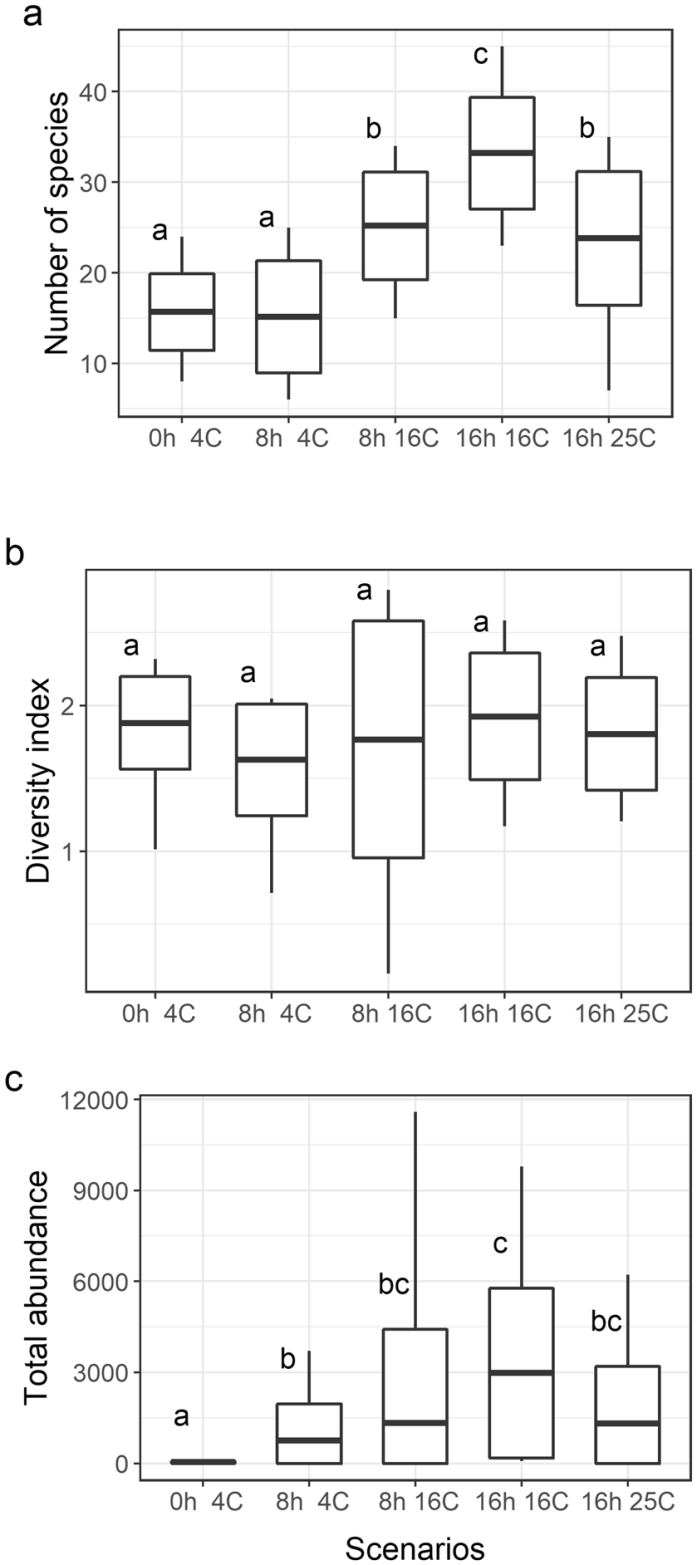
Differences in (**a**) species richness, (**b**) Shannon–Weaver diversity index values, and (**c**) total abundance between the five climate scenarios (whiskers: minimum and maximum values, box: standard deviation, and line: mean; N = 75). The same letters at the boxes denote lack of significant difference (*P* ≥ 0.05) between particular groups according to a post-hoc test. The graph was created in R 4.0.4 under RStudio 1.4.1106 using the ‘ggplot2’ package and the data in S1.

Among the five climate scenarios analysed in the present paper, species richness was the lowest under the 4 °C/0 h and 4 °C/8 h scenarios, whereas the highest values were recorded under the 16 °C/16 h scenario (Fig. 1a). These differences were significant at F_4,70_ = 15.48, *P* < 0.001.

**Figure 2:**
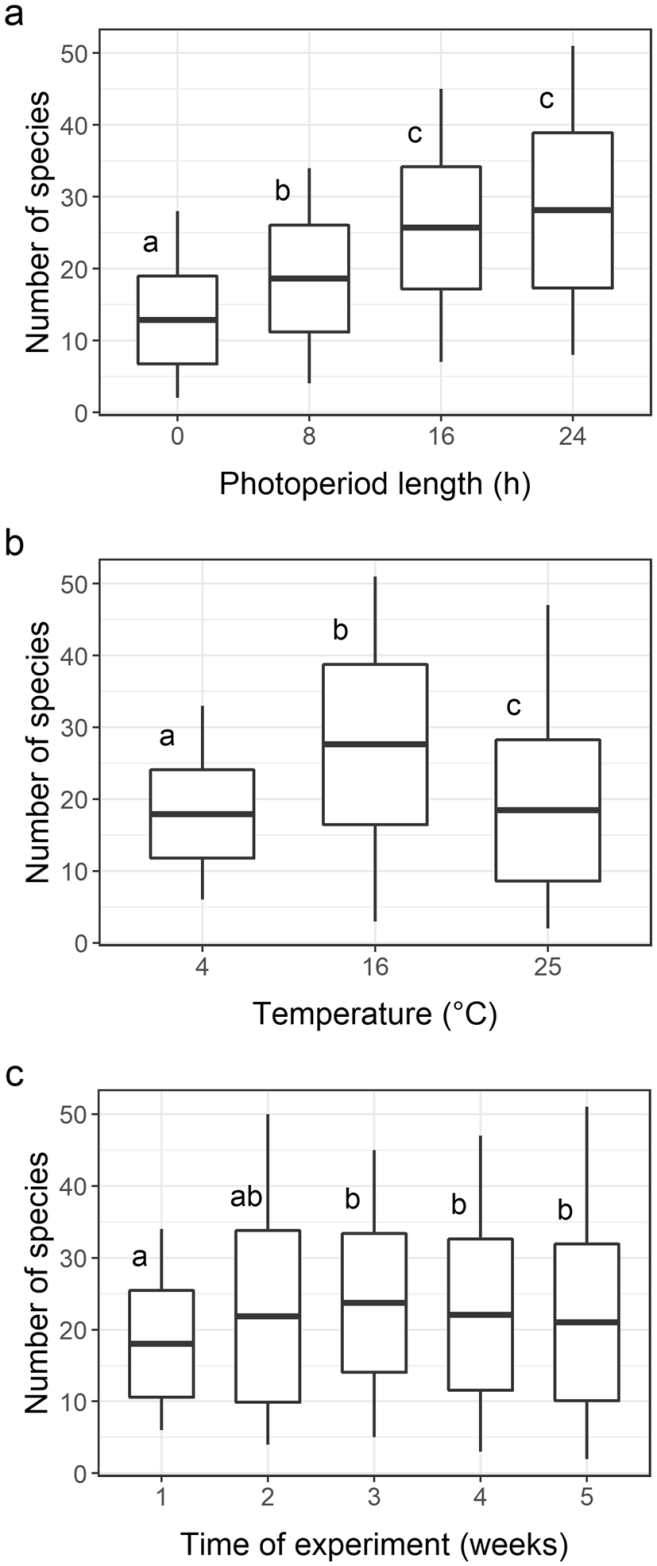
Phytoplankton species richness: (**a**) at different photoperiod lengths, (**b**) at different temperatures, and (**c**) in successive sampling weeks (1–5). The same letters at the boxes denote lack of significant difference (*P* ≥ 0.05) between particular groups according to a post-hoc test (whiskers: minimum and maximum values, box: standard deviation, and line: mean; N = 180). The graph was created in R 4.0.4 under RStudio 1.4.1106 using the ‘ggplot2’ package and the data in S1.

When analysing all the treatments, a significant influence of the interaction between photoperiod length and temperature on the total number of taxa was found (F_6,120_ = 5.362; *P*_adj_ = 0.0003). Light conditions alone were significant at F_3,120_ = 34.458; *P*_adj_ = 0.0003; the longer the photoperiod was, the higher the number of phytoplankton taxa (Fig. 2a). The influence of temperature was also significant at F_2,120_ = 23.08; *P*_adj_ = 0.0003 (Fig. 2b). Among the temperatures, 16 °C resulted in the greatest taxonomic richness, regardless of photoperiod length. Nevertheless, at 16 °C, when the photoperiod was long (16 h and 24 h), of the groups, chlorophytes were the most taxon-rich group, whereas at shorter photoperiods and the same temperature, diatoms and euglenoids were the two groups with the highest number of taxa (see data in S1 and S2.1 for a more detailed description of the successional sequence of particular phytoplankton groups).

Changes in species richness over the time of experiment depended both on light conditions and temperature, as shown by the significance of the interactions between photoperiod and time (F_12,120_ = 1.600; *P*_adj_ = 0.0466) as well as those between temperature and time (F_8,120_ = 2.314; *P*_adj_ = 0.0065). The general model showed that during the experiment, the total number of taxa significantly increased (F_4,120_ = 2.565; *P*_adj_ = 0.0178; Fig. 2c). At the level of particular scenarios, however, a significant relation between time and species richness was recorded only for the treatment maintained at 4 °C with an 8 h photoperiod (increase at F_4,10_ = 10.546; *P*_adj_ = 0.0402).

### Changes in the Shannon–Weaver diversity index

**Figure 3:**
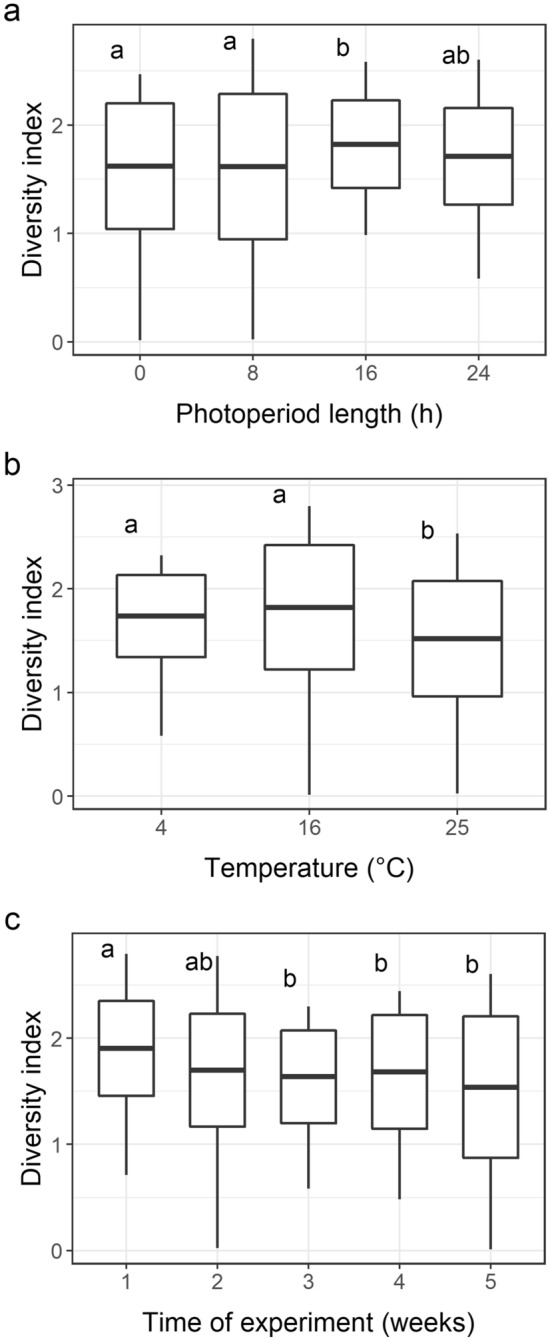
Shannon–Weaver diversity index values: (**a**) at different photoperiod lengths, (**b**) at different temperatures, and (**c**) in successive sampling weeks (1–5). The same letters at the boxes denote lack of significant difference (*P* ≥ 0.05) between particular groups according to a post-hoc test (whiskers: minimum and maximum values, box: standard deviation, and line: mean; N = 180). The graph was created in R 4.0.4 under RStudio 1.4.1106 using the ‘ggplot2’ package and the data in S1.

Both photoperiod and temperature significantly influenced the dynamics of the phytoplankton diversity changes over time (interaction with time: F_12,120_ = 1.7976; *P*_adj_ = 0.0354 and F_8,120_ = 2.4824; *P*_adj_ = 0.006, respectively). When all samples were compared, the values of the Shannon–Weaver diversity index significantly decreased over the time of the experiment at F_4,120_ = 2.1340 and *P*_adj_ = 0.0356 (Fig. 3c). The diversity among the treatments conducted under particular temperature regimes also differed significantly (F_2,120_ = 4.6745; *P*_adj_ = 0.0012). The highest mean value (2.423) of the index was noted at 16 °C, and the lowest mean value (0.764) was observed at 25 °C (Fig. 3b and data in S1). The values of the Shannon–Weaver diversity index, on the other hand, were not significantly different among the treatments differing in the photoperiod length (F_3,120_ = 2.1165; *P*_adj_ = 0.056; Fig. 3a); there were also no differences with respect to these values when the five scenarios were compared (F_4,70_ = 1,375; *P* = 0.194; Fig. 1b). For a more detailed description of temporal changes in the Shannon–Weaver diversity index values in particular treatments, see S2.2.

### Effect of temperature and photoperiod on phytoplankton abundance

**Figure 4:**
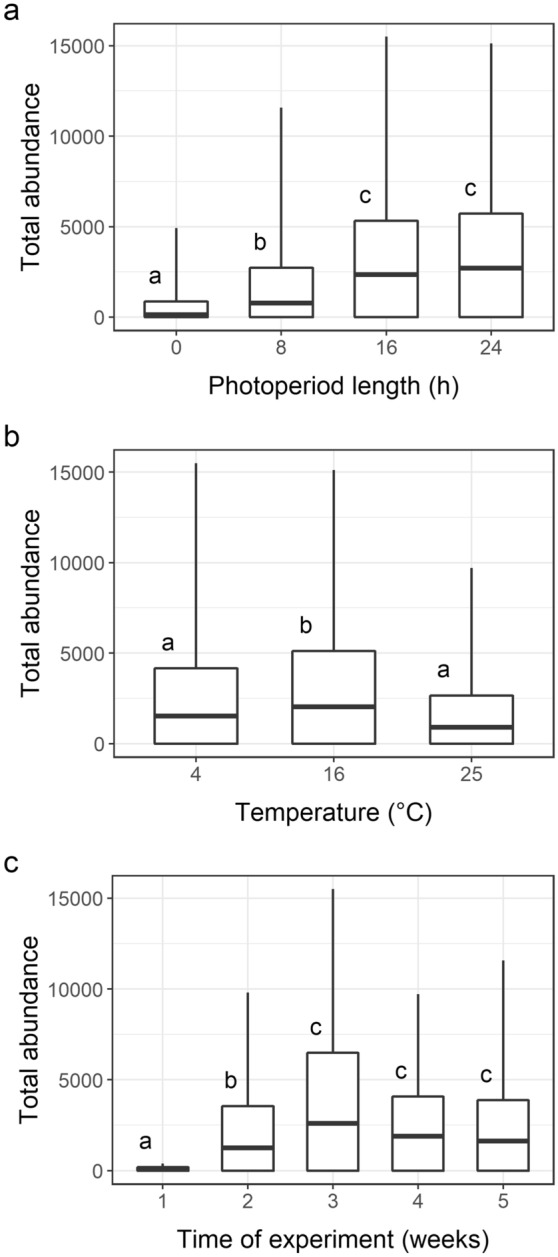
Phytoplankton abundance: (**a**) at different photoperiod lengths, (**b**) at different temperatures, and (**c**) in successive sampling weeks (1–5). The same letters at the boxes denote lack of significant difference (*P* ≥ 0.05) between particular groups according to a post-hoc test (whiskers: minimum and maximum values, box: standard deviation, and line: mean; N = 180). The graph was created in R 4.0.4 under RStudio 1.4.1106 using the ‘ggplot2’ package and the data in S1.

Photoperiod and temperature interactively influenced phytoplankton abundance (F_6,120_ = 2.1561 and *P*_adj_ = 0.0067). Both factors significantly influenced the total phytoplankton abundance (F_3,120_ = 28.7511; *P*_adj_ = 0.0003 and F_2,120_ = 3.0773; *P*_adj_ = 0.0087). There was also a significant impact of photoperiod and temperature on the changes in phytoplankton abundance over time (interaction time x photoperiod: F_12,120_ = 2.4577; *P*_adj_ = 0.0006; time x temperature: F_8_,_120_ = 2.2478; *P*_adj_ = 0.006; for details see data in S2.3). As expected, in all of the temperature treatments, the longer the photoperiod was, the higher the mean total phytoplankton abundance was (Fig. 4a). Among the temperatures, 16 °C had the highest values of abundance, and the lowest abundance was noted at 25 °C (Fig. 4b). In general, the abundance increased until the fourth week of the experiment and then stabilised; the differences depending on time alone were significant at F_4,120_ = 8.6733 and *P*_adj_ = 0.0003 (Fig. 4c).As expected, of the photoperiods, the 0 h photoperiod resulted in the lowest number of phytoplankton individuals, and the number remained stable during consecutive samplings, regardless of temperature (F_4,40_ = 1.4216; *P*_adj_ = 0.089). At the 8 h photoperiod, the total abundance of phytoplankton gradually increased over time (F_4,40_ = 3.2912; *P*_adj_ = 0.004) and consistently reached the highest values in the last week of the experiment. At the 16 h photoperiod, the abundance increased over time until the third week (with cryptophytes being dominant at 4 and 25 °C and chlorophytes at 16 °C) before decreasing (F_4,40_ = 4.8077; *P*_adj_ = 0.004). At the 24 h photoperiod, phytoplankton abundance increased until the 3rd or 4th week (with cryptophytes dominant at 4 °C and chlorophytes at 16 °C) before decreasing (F_4,40_ = 4.8277; *P*_adj_ = 0.004).

The differences in abundance were also significant under specific climate scenarios (F_4,70_ = 5.6144; *P* = 0.001), with lowest values recorded under 4 °C/0 h and highest under 16 °C/16 h (Fig. 1c). The increase in abundance over the time of the experiment under specific climate scenarios was significant in the case of the treatment maintained at 4 °C and the 8 h photoperiod (F_4,10_ = 1.0155; *P*_adj_ = 0.0258).

### Response of phytoplankton taxonomic groups to particular experimental treatments

Chlorophytes, diatoms and cryptophytes were the major dominant groups, whereas chrysophytes occurred in only one sample. For a detailed description of the temporal changes in abundance and in the share of the particular phytoplankton groups in the total phytoplankton abundance in the different treatments, see data in S1 and S2.3.

The changes in abundance over time were significant in the case of chlorophytes (F_4,120_ = 5.6897; *P*_adj_ = 0.0007). The abundance of this group increased until the 4th week, with significant differences between the first two samplings and all the remaining samplings (F_1,48_ > 3.9; *P*_adj_ < 0.015). There was no interaction, however, between time and photoperiod (F_8,120_ = 0.7697; *P*_adj_ = 1.0000) or time and temperature (F_12,120_ = 1.7989; *P*_adj_ = 0.2148) in the case of chlorophyte abundance or in the case of any remaining group (F < 2.1; *P*_adj_ > 0.16).

The interaction between photoperiod and temperature was significant only in the case of dinoflagellates (F_6,120_ = 5.1499; *P*_adj_ = 0.0014). Photoperiod and temperature were also significant for this group when analysed alone (F_6,120_ = 5.1499; *P*_adj_ = 0.0014 and F_6,120_ = 5.1499; *P*_adj_ = 0.0014, respectively). The significant interaction was caused by the fact that the abundance of dinoflagellates was almost limited to the treatments with longer photoperiods (F_1,48_ > 4.6, *P* < 0.02), whereas their abundance was the highest in treatments maintained at 25 °C (F_1,48_ > 9.8; *P* < 0.003).

The light climate was the sole factor influencing the abundance of the cryptophytes (F_3,120_ = 6.4777; *P*_adj_ = 0.0014). The abundance of this group was the highest under 16 h and 24 h photoperiods (differences between all the groups were significant at F_1,48_ > 6.5; *P* < 0.008, except for 16 h vs 24 h: F_1,48_ = 0.0247; *P* = 0.889). Temperature, on the other hand, was the only factor significant for cyanobacteria (F_2,120_ = 4.1470; *P* = 0.038) and diatoms (F_2,120_ = 5.0925; *P* = 0.0174). The first group reached the lowest mean abundances at 25 °C and the highest at 16 °C, with significant differences between treatments maintained at 4 °C versus 25 °C (F_1,48_ = 3.3253; *P* = 0.002) and 16 °C versus 25 °C (F_1,48_ = 6.6219; *P* = 0.001). A similar situation occurred in diatoms, with significant differences when comparing 4 °C versus 16 °C (F_1,48_ = 4.3366; *P* = 0.023) and 16 °C versus 25 °C (F_1,48_ = 6.1319; *P* = 0.005).

**Figure 5:**
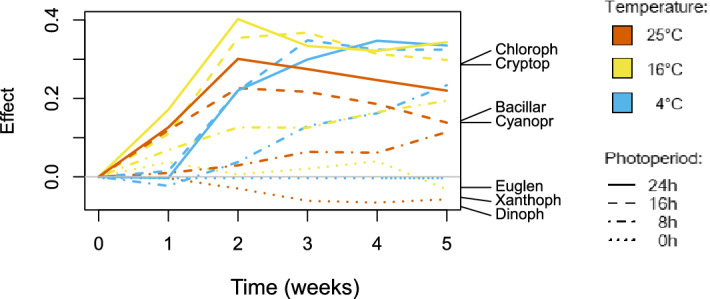
Principal response curves (PRC) illustrating changes over time in the structure of the phytoplankton communities in particular experimental treatments. Secondary axis (right): first canonical axis of the Redundancy Analysis performed on the abundances of particular taxonomical groups. The graph was created in R 4.0.4 under RStudio 1.4.1106 using the ‘vegan’ package and the data in S1. Phytoplankton taxonomic groups/abbreviations: *Cyanopr* - Cyanoprokaryota (cyanobacteria), *Chloroph* - Chlorophyta (chlorophytes), *Cryptop* - Cryptophyta (cryptophytes), *Bacillar* - Bacillariophyceae (diatoms), *Euglen* - Euglenophyta (euglenoids), *Dinoph* - Dinophyceae (dinoflagellates), *Xanthoph* - Xanthophyceae (xanthophytes).

The relations mentioned above were reflected by the results of the Principal Response Curves (PRC) analysis (Fig. 5). According to the initial analysis, throughout the experiment, the communities could transform towards three general directions, hereafter referred to as three community types: (1) chlorophyte or cryptophyte dominance; (2) increasing diatom and cyanobacteria abundance; and (3) euglenoid-xanthophyte-dinoflagellate dominance. This partitioning was illustrated by the first canonical axis of the Redundancy Analysis (RDA) (eigenvalue = 9.259, significant at F_1,168_ = 298.89, *P* < 0.001), which was subsequently used for the analysis of trends applying the PRC analysis (Fig. 5, right side of the graph). The second RDA axis was not significant (eigenvalue = 1.043, F_1,168_ = 33.656, *P* = 0.416); thus, we did not use it for the PRC analysis.

According to the resulting graph, specific experimental treatments grouped well with respect to the photoperiod lengths (marked with different line types in Fig. 5). The treatments with the longest (24 h, solid lines) and the second-longest (16 h, dashed lines) photoperiods displayed very similar patterns. Their phytoplankton communities were quickly dominated by chlorophytes and cryptophytes (type 1). In the case of those in which temperature was the highest, however, there was a visible turning point after the second week of the experiment, and the communities turned towards diatom dominance accompanied by cyanobacteria (type 2). Communities in the lines with the 8 h photoperiod (dash-and-dotted lines) slowly and gradually transformed from type 3 communities towards type 2 communities. The treatments from the control groups, maintained in the dark for the entire period, corresponded to type 3, with no visible change over time (dotted line). No such clear pattern grouping the experimental lines was visible if the treatments were arranged with respect to temperature (the same line colours, Fig. 5), except for those maintained at the highest temperature (red lines, Fig. 5). Although the trajectory of the changes was different for each of these lines, all seemed to aim at diatoms/cyanobacteria as their final community type (except for the line maintained in the dark, dominated by the type 3 community). The model was significant at F_55,120_ = 4.0851, *P* < 0.001.

### Species-level response of phytoplankton communities to experimental conditions

**Figure 6:**
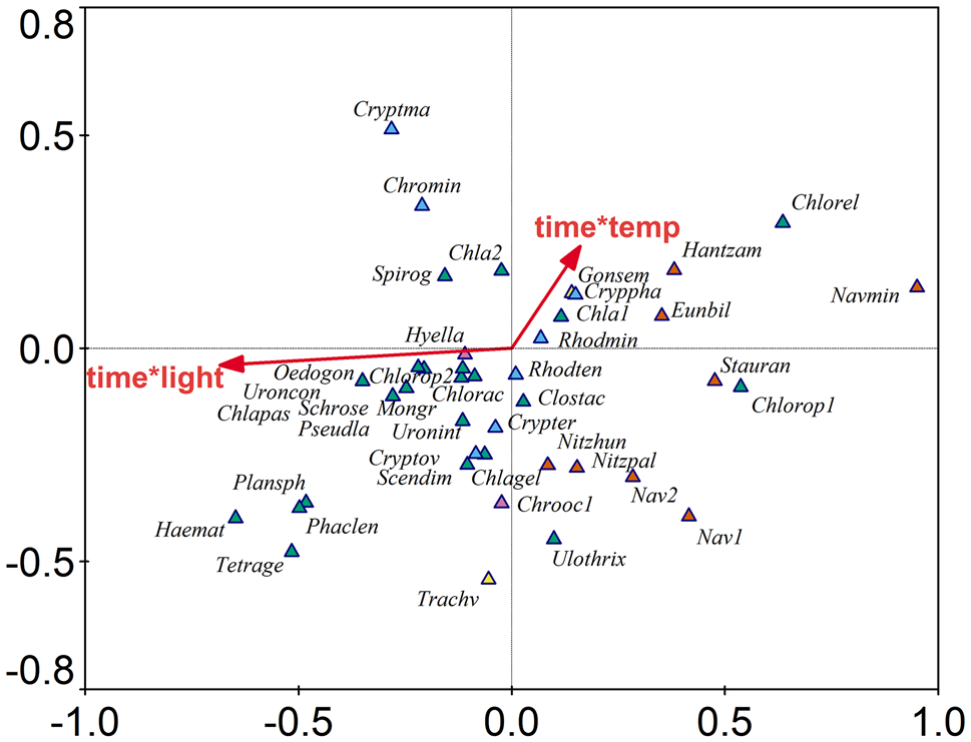
Results of CCA illustrating the phytoplankton species distribution over time and the environmental variables (time*temp—temperature over time, time*light—photoperiod length over time). For abbreviations see S2.4. The graph was created using the Canoco 4.56 software package and the data in S1.

Species-level Canonical Correspondence Analysis (CCA) was performed on the 41 most dominant and frequent species (Fig. 6). Most of these species belonged to chlorophytes, diatoms and cryptophytes (22, 8 and 7 taxa, respectively). The model showed that changes in the structure of the phytoplankton communities at the species level were significantly influenced by both temperature (F = 4.65; *P* = 0.001) and photoperiod length (F = 7.43; *P* < 0.001). According to the results, changes in abundance over the time of the experiment for some chlorophytes (*Spirogyra* sp. and *Chlamydomonas sp.* 2) and cryptophytes (*Cryptomonas marssonii* and *Chroomonas minuta*) were positively associated with both photoperiod and temperature, while diatoms (*Nitzschia hungarica, N. palea, Navicula* sp*.,* and *Stauroneis anceps* f. *gracilis*) were negatively affected by these parameters. The dynamics of a large group of species (mostly chlorophytes: *Oedogonium* sp., *Uronema intermedium, U. confervicolum*, *Haematococcus pluvialis*, *Monoraphidium griffithii*, *Schroederia setigera*, *Planctococcus sphaerocystiformis*, *Phacotus lenticularis, Pseudosphaerocystis lacustris, Chlamydomonas passiva, Tetraspora gelatinosa,* and *Chlorogonium elongatum* var*. aculeatum*) were positively correlated with photoperiod length and negatively correlated with temperature. Changes in the abundance of diatoms (*Hantzschia amphioxys*, *Eunotia bilunaris,* and *Navicula minima*) and the chlorophyte *Chlorogonium elongatum* were negatively correlated with photoperiod length only. Moreover, the abundances of *Cryptomonas phaseolus, Chlamydomonas* sp. and *Euglena* sp. increased over time in the treatments at higher temperatures. The whole model was significant: F = 6.117; *P* < 0.001; significance of the first canonical axis was F = 7.396; *P* < 0.001; and the eigenvalue of the first axis was 0.238, and that of second axis was 0.146.

## Summary and discussion

Our results indicate that vernal pool phytoplankton communities respond differently to particular climate change scenarios related the temperate climate zone. The selected combinations of temperature and photoperiod length significantly affected phytoplankton succession and community structure at the species richness and abundance levels.

**Figure 7:**
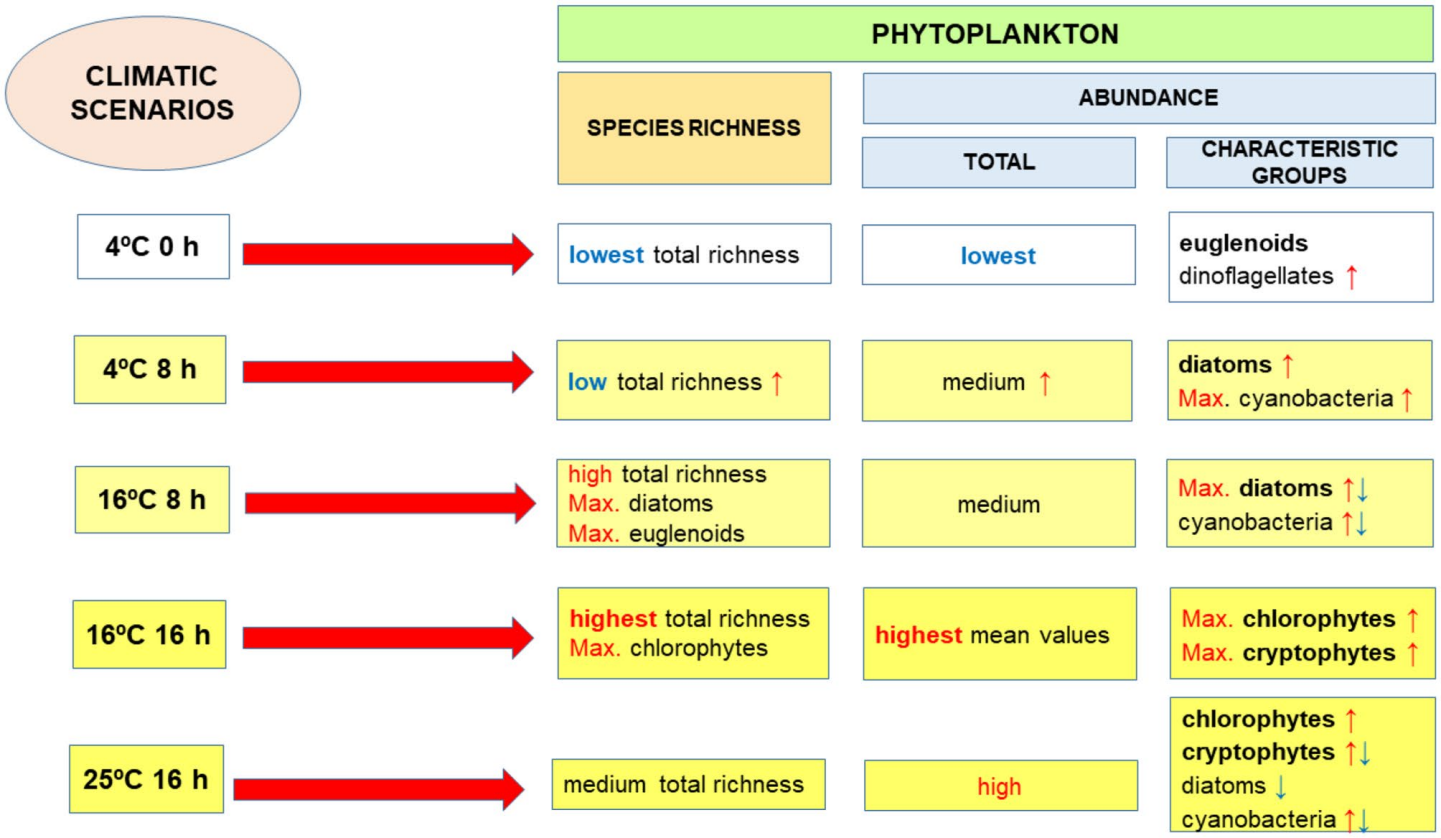
Summary of the effects of different climate scenarios on phytoplankton communities (species richness, Shannon–Weaver diversity index values and abundance) over time. Legend: ↑—increase, ↓—decrease, bold: dominant groups. The graph was created using MS PowerPoint software.

Among the five climate scenarios that we took into account in the experiments, two (4 °C, photoperiod 0 h and 4 °C, photoperiod 8 h) reflected the present climate conditions and corresponded to our field observations (see S3). The first scenario (4 °C, photoperiod 0 h) is typical for vernal pools during cold and snowy winters. Ponds fill with water from transient snowmelt in February, and then, the surface freezes and becomes covered with snow, blocking sunlight access for phytoplankton. Such conditions in general inhibited photosynthetic microalgal growth in our experiments, causing both low values of the total number of taxa and individuals (Fig. 7). Over time, the phytoplankton community transformed towards euglenoids and dinoflagellates, reflecting our field observations (compared with data in S3). In particular, euglenoids accounted for a high share of the total abundance of phytoplankton communities in the experimental microcosms and in the natural vernal pool in winter soon after inundation, even when the water surface froze. This group of algae is known to live within a wide range of temperatures (also confirmed in our research); therefore, photoperiod seemed to be the major factor affecting their abundance in this scenario.

The second climate scenario (treatment: 4 °C, photoperiod 8 h) often occurs in temperate climate zones under current climate change conditions, when pools become inundated in February as a consequence of snow thawing or rains but do not freeze (see S3). Similar to the results of the previous scenario, a low total number of phytoplankton was noted under this scenario (Fig. 7). The species richness and total abundance of phytoplankton, however, significantly increased in this treatment, and communities transformed towards type 2, where diatoms and cyanobacteria were the characteristic groups. This result is in accordance with our field observations, where diatoms often dominated in the phytoplankton communities immediately after inundation of the vernal pool, during a short photoperiod in winter or early spring, from which the sediments for our experiments were taken (see data in S3).

The results obtained under the climate scenario with a very short, wet winter and a sudden increase in temperature (16 °C, 8 h photoperiod) that was predicted for the future with progressive warming were similar to those obtained under the previous scenario, and the community trended in the same direction – towards diatoms and cyanobacteria. The overall algal abundance was also similar between the two scenarios; however, the number of species was significantly higher under this scenario than under the previous scenario. The increase in future winter temperatures could therefore increase phytoplankton species richness in vernal pools, promoting the development of diatoms, euglenoids and cyanobacteria. Such a change would certainly influence ecosystem functioning, e.g., by increasing the abundance of filter feeders. On the other hand, increasing algal abundance could reduce the development and productivity of macrophytes as a result of competition for nutrients and light by primary producers, substantially transforming the special structure of the ecosystem.

Another climatic scenario with a moderate temperature (16 °C) and a long photoperiod (16 h) predicted a dry winter and a mild, rainy spring in the future, when the ponds would fill later in the season (late spring inundation with medium temperatures). Such conditions promoted phytoplankton species richness, similar to the above scenario with the same temperature but a short photoperiod (Fig. 7). Among the scenarios, this scenario resulted in the total algal abundance being the highest, the dynamics of the changes being more rapid from the start of the water phase, and the direction of the changes being different. The phytoplankton community in the case of this scenario trended towards type 3 with chlorophyte and cryptophyte dominance. Thus, if the climate change conditions predicted under this scenario occur, then the early structure of phytoplankton communities will be almost entirely changed, and the influence of these changes on the functioning of the whole ecosystem will probably be even more pronounced than in the case of the previous scenario.

The climate scenario with the highest temperature (25 °C) and long photoperiod (16 h) predicted a dry winter with a hot late spring (or onset of summer) with heavy showers. Under such conditions, both species richness and abundance were relatively high (abundance, however, was significantly lower than that in the 16 °C/16 h scenario). The trend in the changes in community structure was between types 2 (diatoms-cyanobacteria) and 3 (chlorophytes-cryptophytes). This trend resulted from the fact that although in the later stages of the experiment the chlorophytes dominated quantitatively, the share of cyanobacteria was relatively high when compared to that in the other treatments. Nevertheless, the cyanobacteria were far from dominant: they reached a maximum abundance of 216 individuals/mL during the fourth sampling, while at the same time, the abundance of chlorophytes was 3828 individuals/mL. Moreover, the abundance of cyanobacteria was much higher under the 4 °C/8 h scenario (1428 individuals/mL) than under the other scenarios. The results of our experiments disprove the hypothesis that this group will become dominant in vernal pools with an increase in water temperature. This result is surprising and inconsistent with those in many other studies (conducted on permanent water bodies), which showed that cyanobacteria have a high growth potential at elevated temperatures^40–44^ ranging from 20 to 35 °C^19^ and that they usually increase their abundance and biomass with global warming^26,32,45^. According to our study, vernal pools seem to be inhabited by specific cyanobacterial species that prefer lower water temperatures, especially soon after inundation (during the initial stages of phytoplankton succession).

Our general analyses conducted on all the treatments showed that the Shannon–Weaver diversity index depended significantly on temperature but not photoperiod. In the course of succession, high temperatures favoured a decline in species diversity. These findings were in line with our initial hypothesis and with the findings of many earlier studies^21,46–50^, which also showed that phytoplankton species diversity decreased with higher temperatures (with climate warming). On the other hand, species richness and abundance depended on both temperature and photoperiod. For abundance, however, both at the level of the whole community and particular taxonomic groups, light conditions seemed to be the most important factor, as emphasised by our PRC analyses. The combination of these observations suggests that temperature could be the factor that determines phytoplankton species diversity, while photoperiod could be the factor that determines the quantitative structure of the community. The influence of both factors together exerted an influence on the changes in phytoplankton diversity over the course of succession and determined the trajectory of the observed changes.

Some phytoplankton taxa are known for coping with extended periods of darkness (e.g., in polar regions or buried in sediments) by using a diverse range of strategies, such as reducing their viability, producing resting spores, reducing their metabolic rates or using stored energy reserves^3,51^. Many flagellated species (e.g., euglenoids and dinoflagellates) are able to survive in the dark due to mixotrophy as a nutritional strategy^52^, and in the absence of light, they feed on dead organic matter. Moreover, euglenoids, dinoflagellates and xanthophytes, as flagellated forms, may easily move through the water column to surface layers for more light if there is access to it. In this way, their resistance to light deficiencies gives them a competitive advantage over strictly photoautotrophic microalgae. Such a relation was also visible in our experiments: under dark conditions (simulated light conditions in winter under ice in vernal pools in the temperate climate zone), the phytoplankton communities transformed mainly towards mixotrophic and flagellated euglenoids and dinoflagellates.

According to the CCA analysis, photoperiod length negatively affected temporal changes in the abundance of all dominant diatom taxa from the microcosms, some of which were also under a similar influence of temperature. Moreover, in all studied microcosms (under 12 combinations of temperatures and photoperiods), diatoms initiated the successional process, regardless of the climate scenarios. Diatoms are often the pioneering components of phytoplankton communities in vernal pools in the temperate zone (see S3), being dominant at the onset of the hydroperiod. They are generally known to tolerate cold environments^53^ and to survive under low light intensity^54^, and vegetative stages of benthic taxa are able to survive for months of darkness^55^. This ability is associated with a reduction in their metabolic rate^3^, giving diatoms a competitive advantage in regions with a prolonged absence of irradiance^55^. The same ability makes them successful competitors at the onset of the hydroperiod in vernal pools under natural conditions (short day and low temperature), as our study shows. Moreover, diatoms are known for their fast reproduction and short life cycles^56^, additionally explaining their initial dominance and rapid decline after the first week of the investigations, regardless of photoperiod. In the future, if vernal pools in temperate climate zones become inundated later in the season (lack of snow cover and thus no surface runoff connected with thawing), diatoms may experience strong competitive pressure due to progressive warming because of the longer daytime favoured by other groups. Thus, the phytoplankton community structure will change significantly.

Similar to diatoms, cyanobacteria in our experiment preferred short photoperiods and low (4 °C) or moderate (16 °C) temperatures (Fig. 7). Some representatives of this wide taxonomic group are known for low light requirements^57,58^, which could grant them a competitive advantage over most algal groups (except diatoms), as observed in our short photoperiod treatments. However, the abundance and share of cyanobacteria in the total community compared with those of the diatoms were always very small in the microcosms. Moreover, at the initial stages of succession in our original vernal pool, there was even a lack of cyanobacteria (see S3). A deficiency in nutrients could be one of explanations: there is no substantial inflow of nutrients in vernal pools soon after inundation (in the initial stage of phytoplankton succession), until agricultural activities start in the mid-spring, and neither condition occurred in our experimental microcosms. Therefore, cyanobacteria could be lost due to competition with other species typical of small water bodies. Euglenoids or fast-growing single-celled chlorophytes, cryptophytes and originally benthic or periphytic diatoms tolerate water mixing and lower nutrient concentrations characteristic of the initial stage of succession. Thus, resting spores of such species adapted to specific conditions of temporary ponds prevail in the spore bank stored in the sediments, and the only temporal niche for cyanobacteria occurs under a short daytime if the other conditions are favourable.

With long photoperiods (scenarios predicting later inundation of ponds in the future in the temperate climate zone, with progressive global warming), phytoplankton communities transformed towards chlorophytes and cryptophytes. These two most abundant microalgal groups (dominated by small, fast-growing r-strategists, typical in temporary waters^20^) should be a preferred food source for grazers (zooplankton) in vernal pools. Feeding studies with natural phytoplankton mixtures have confirmed that chlorophytes (especially the flagellates dominant in our microcosms) and flagellated cryptophytes are favoured foods for zooplankton^59^. These algae are supposed to be easy to graze upon because the cells by their flagella may adhere to the feeding appendages of zooplankton and because they do not have sturdy cell walls. Therefore, late inundation of ponds in the future may promote the development of zooplankton populations (provided with plentiful and preferable food resources) and hence the development of zooplankton consumers, e.g., amphibians.

Among all climate change scenarios, long photoperiods and moderate temperatures seem to be the most favourable conditions for microalgae. Such conditions favoured not only the development of easily eaten chlorophytes and cryptophytes but also the total species richness and abundance of phytoplankton. Therefore, the scenarios predicting progressive global warming in temperate zones should stimulate zooplankton development in vernal pools, which in turn may lead to a reduction in algal abundance. On the other hand, zooplankton may not only regulate microalgal abundance by grazing but also stimulate their growth through the efficient recycling of nutrients (as a result of nutrient excretion) that are essential for phytoplankton growth^60,61^. Moreover, algae under strong grazing pressure are able to increase their abundance by their rapid growth rates that compensate for increased grazing losses^62^. Thus, in the long term, zooplankton grazing pressure may contribute to the persistence of high phytoplankton abundance, which in turn will inhibit macrophyte growth at moderate temperatures during late spring inundation in vernal pools.

The microalgae in our experiment were obtained from the sediments of the temperate climate vernal pool, and they usually desiccate before the average water temperature exceeds 16 °C. Thus, resting cells of species adapted to the lower temperatures of the spring season prevail in the sediments, explaining the observed decrease in abundance and species richness at the highest temperature. Under the global warming scenarios predicting an increase in spring temperatures and heat wave frequencies, the pioneering species at the beginning of the water phase could be threatened. Entire algal communities, including the species dominating in the late phases of the hydroperiod, will be out of the range of their temperature optima. Such a disturbance will vastly influence the functioning of the whole ecosystem, which is largely dependent on phytoplankton and invertebrate filter feeders^20^.

Under optimistic circumstances, if climate changes in the central European temperate zone cause a local lowering of summer temperatures, as predicted by some scenarios, the pioneering species might also have the potential to be dominant in early summer if the hydroperiod extends. On the other hand, an increase in the temperatures (as predicted by most of the models) could be seen as conserving a high portion of the present species diversity related to later stages of succesion^63^. Nevertheless, the most typical diversity of the vernal pools related to lower temperatures (promoting diatoms most of all) will be lost. To put it bluntly, the pools will be vernal no more. This study, conducted in vernal pool microcosms, enriches the knowledge about the functioning of temporary waters and is a valuable model for a broad range of ecological investigations and monitoring in the era of global climate change. Knowledge of the photoperiod and temperature preferences of phytoplankton and the course of succession under different scenarios is essential for predicting how primary producers and aquatic ecosystems will respond to future climate warming. Our results highlight threats to vernal pools under global warming scenarios predicting rising temperatures: seasonal shifts in species abundances (or even their disappearance) and decreases in species diversity, which will strongly affect the food webs of aquatic environments in the future.

## Methods

**Figure 8:**
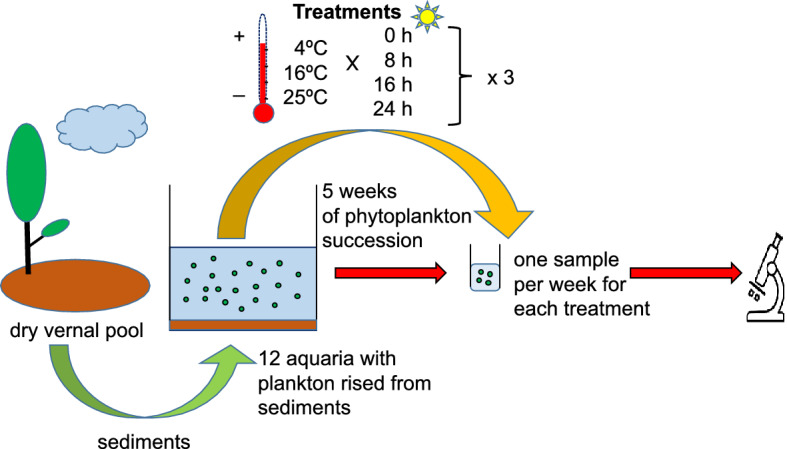
Successive stages (steps) of investigations: from the field through the experimental design/laboratory experiments in microcosms. The graph was created using MS PowerPoint software.

At the beginning of the experiment (Fig. 8), we collected a sample of bottom sediments from a temporary pond located in western Poland (52° 29′ 02″ N; 16° 37′ 08″ E). This pond is one of the vernal pools forming a cluster of ponds in this area (see^64^ for a map and some basic parameters) and is usually inundated in February (length of the day: 9 h; mean temperature: − 1.1 °C) with water from thawing snow. The water phase (maximum depth: 1.2 m) lasts for an average of four months, and the pond desiccates completely in late May/June (length of the day: 16 h; mean temperature: 15.1 °C). The sediment sample was collected in August from the dry bottom of the pond, which was covered at this time by monocots (mainly *Agrostis stolonifera*). The sample was formed of a series of approximately 40 subsamples of the top 6 cm layer of sediments collected at random areas scattered evenly over the surface of the pond.

After being transported to the laboratory, the sediments were sieved using a 5 mm soil sifter before being mixed and homogenised. The resulting material (36 L of sediments) was divided into three parts that were used for subsequent repetitions of the experiment. For each repetition, we used 12 glass aquaria, each filled with 1 L of sediments and 10 L of deionised water as a substitute for melted snow water or rainwater.

The aquaria were stored for 5 weeks in three rearing rooms with constant temperatures (4, 16 or 25 °C), with four aquaria in each room. Three aquaria in each set were equipped with a 6500 K, 900 lm cold light source set for an 8, 16 or 24 h photoperiod. One aquarium in each room was left in the dark as a control (at 4 °C to simulate conditions prevailing under ice when the vernal pools freeze after initial inundation). The sediments for the three subsequent repetitions were stored dry in the dark at + 4 °C.

The experimental design of our study aimed to simulate vernal pool environments under five climate scenarios (two present/recent and three future) according to prediction models for central Europe^65–67^: (1) a cold, snowy winter where ponds fill with water from transient snowmelt in February, and then, the surface freezes—a scenario typical for vernal pools, still occurring currently and prevailing some 10–20 years ago (treatment: 4 °C, photoperiod 0 h); (2) a mild, wet winter where pools inundate in February as a consequence of snow thawing or rains but do not freeze (treatment: 4 °C, photoperiod 8 h); (3) a very short, snowy or rainy winter followed by a sudden increase in temperature (spring in February scenario; treatment: 16 °C, photoperiod 8 h); (4) a dry winter and mild, rainy end of the spring (16 °C, 16 h); and (5) a dry winter with a hot spring/summer with heavy showers (25ºC, 16 h). The treatments conducted using a 24 h photoperiod showed the sole influence of temperature when access to light was unlimited.

From each aquarium, weekly samples (1 L) were collected for phycological analyses. Based on the samples, temporal changes in phytoplankton were observed over 5 weeks (for each repetition and each combination of temperature and photoperiod). In total, 12 experimental combinations of temperature and photoperiod were compared, each in 3 replicates of 5 samplings, producing 180 samples. Five experimental combinations (treatments) corresponded to the five climate scenarios mentioned above and the remaining ones resulted from full factorial design needed for testing the influence of interactions between the variables. Water samples for phycological analyses were fixed with Lugol solution. Samples were sedimented in the laboratory, concentrated to a volume of 5 mL, and then preserved with formalin. Phycological investigations were conducted under a light microscope (at 200 ×, 400 × and 1000 × magnification). Phytoplankton cells were counted in a Fuchs-Rosenthal chamber (height: 0.2 mm, area: 0.0625 mm^2^). Unicellular microalgae and colonies were treated as individual units. In the case of trichomes, the length of the individual was standardised. The standard length of the individual was considered 100 μm. In the case of species forming colonies (e.g., *Aphanocapsa* sp. and *Aphanothece* sp.), a cover area of 400 μm^2^ was classified as a unit.

Since the abundance of particular taxa was far from fitting any classical distribution type, we used permutational multivariate analysis of variance (PERMANOVA) to analyse the data. The analyses were performed to check for differences in the total number of taxa, the abundance of phytoplankton and the values of the Shannon–Weaver diversity index between the experimental photoperiod and temperature levels as well as the time variable (particular sampling events). Interactions between both experimental variables, as well as between each of them and time were included in the models, and all the analyses were stratified with respect to the blocks coding three repetitions of the experiment^68^. To account for type I errors due to multiple tests being conducted, Holm's correction was used to adjust the reported *P* values. Post hoc pairwise multilevel comparisons between particular levels of explanatory variables were conducted using the pairwiseAdonis algorithm^69^.

Particular species, characterised by individual preferences and adaptations, respond to environmental factors with different dynamics. Slower or more rapid changes in abundance over the time of the observation are reflected in the sequence of changes in the community structure. To analyse patterns of such changes at the level of particular taxonomic groups, Principal Response Curves (PRC) analysis was used^70^. Patterns at the level of particular species were more complex, so Canonical Correspondence Analysis (CCA) with an interaction between time and treatment was used instead.

PRC was conducted based on partial redundancy analysis^71^: first, Redundancy Analysis (RDA) was conducted on the log-transformed data on the abundance of particular taxonomic groups. Sampling time indicators were used as covariables, and the interactions between the treatment levels and sampling times were used as explanatory variables. The Monte Carlo permutation test (5000 permutations) was used to test the significance of particular variables as well as the first and second canonical axes. Next, canonical scores of explanatory variables from the first canonical axis were extracted and used to draw response curves for each treatment. On the right side of the resulting graph also species scores of the first RDA axis are displayed, enabling interpretation of the direction of departure from the community composition in the reference treatment (4 °C and darkness).

In canonical analyses, records of species with low frequencies are often over-emphasised, unduly influencing the results of ordination^71–73^. To avoid such bias and reduce the noise caused by the stochastic occurrence of some rare species, CCA was conducted only on data of the most numerous and dominant taxa. As such, we considered taxa with a high frequency (occurring in more than 5% of samples, i.e., at least 10 samples) and high abundances (reaching abundances of at least 200 individuals per mL in all the samples). In total, 41 species passed these criteria and were included in the CCA (22 chlorophytes, 8 diatoms, 7 cryptophytes, 2 cyanobacteria and 2 euglenoids). Interactions between particular treatments and samplings (time x photoperiod; time x temperature) were used as explanatory variables, and their statistical significance in the model was assessed using the Monte Carlo permutation test (5000 permutations); the same test was performed on the first canonical axis as well as on the whole model. To avoid pseudoreplication, all of the permutation tests were restricted to blocks of data representing the particular time series analysed (cyclic shifts were used)^72,73^.

Canonical Correspondence Analysis was conducted using the Canoco 4.56 software package^72,73^. The remaining analyses were conducted in R 4.0.4^74^ under RStudio 1.4.1106 using the ‘vegan’ and ‘pairwiseAdonis’ packages^69,75^. We considered *P* = 0.05 as a threshold determining statistical significance in all the analyses.

## Supplementary Information

Supplementary Legends.

Supplementary Table S1.

Supplementary Information.

Supplementary Table S3.
