# Supplementary material for: Phytoplankton communities in temporary ponds under different climate scenarios
Source: Sci Rep. 2021 Sep 9;11:17969. doi: 10.1038/s41598-021-97516-9 (PMC8429430; doi:10.1038/s41598-021-97516-9)
Supplement: Supplementary file 1 — Supplementary Legends. [file 41598_2021_97516_MOESM1_ESM.docx]

**Supplementary Information**

Phytoplankton communities in temporary ponds under different climate scenarios

Sofia Celewicz^1*^, Bartłomiej Gołdyn^2^

^1^Department of Botany, Faculty of Agronomy, Horticulture and Bioengineering, Poznań University of Life Sciences, Wojska Polskiego 71 C, 60-625 Poznań, Poland

^2^Department of General Zoology, Faculty of Biology, Adam Mickiewicz University in

Poznań, 61-614 Poznań, Poland

Corresponding author*:

E-mail: sofia.celewicz@up.poznan.pl

^1*^, ^2^  these authors contributed equally to this work

**SUPPLEMENTARY INFORMATION LEGENDS**

S1. Taxonomic composition, abundance [individuals/mL] and Shannon-Weaver diversity index of phytoplankton in the particular samples (180 in total).

S2.1. The successional sequence of phytoplankton groups based on qualitative analysis.

S2.2. Temporal changes in Shannon-Weaver diversity index values in particular treatments. S2.3. Temporal changes in percentage contributions of phytoplankton groups to the total mean phytoplankton abundance in particular experimental treatments and in the field (vernal pool in winter, in the initial stage of phytoplankton succession).

S2.4. Dominant taxa and their abbreviations on the CCA diagram.

S3. Data from the vernal pool, from which the sediments for experiments were taken: S-W - Shannon-Weaver diversity index values, total phytoplankton abundance [individuals/mL], number of taxa and abundance of the phytoplankton taxonomic groups, abundance of the dominant taxa, dry period, and physical-chemical parameters of water – conductivity [µS cm^-1^], pH, temperature [ºC], N-NH4 [mg/L], N-NO2 [mg/L], N-NO3 [mg/L], SRP – soluble reactive phosphorus [mg/L], TP – total phosphorus [mg/L]; precipitation; average temperature [ºC].
