# Supplementary material for: Phytoplankton communities in temporary ponds under different climate scenarios
Source: Sci Rep. 2021 Sep 9;11:17969. doi: 10.1038/s41598-021-97516-9 (PMC8429430; doi:10.1038/s41598-021-97516-9)
Supplement: Supplementary file 3 — Supplementary Information. [file 41598_2021_97516_MOESM3_ESM.docx]

**2.1. The successional sequence of phytoplankton groups based on qualitative analysis**

In the initial stage of succession (first and/or second week of investigations), the most numerous were always the representatives of diatoms (the most frequent were: *Eunotia bilunaris, Hantzschia amphioxys, Nitzschia palea, Pinnularia mesolepta, Pinnularia viridis, Stauroneis anceps f. gracilis, Stauroneis phoenicentron*) and euglenoids (the most frequent was *Trachelomonas volvocinopsis*), regardless of the photoperiod and temperature. In the subsequent weeks however, the representatives of chlorophytes became the most numerous in the taxa and their participation in the qualitative structure of phytoplankton increased, especially for the photoperiods of 16 h and 24 h.

**2.2. Temporal changes in Shannon-Weaver diversity index values in particular treatments**

The values of the index varied between 0.013 and 2.795. The highest mean value was noted at the 8 h photoperiod and temperature of 16°C in the first week of the experiment, while the lowest mean value was found at the 0 h photoperiod and a temperature of 25°C in the last week. At the temperature of 4°C, a relatively slight fluctuation over time in the values of Shannon-Weaver diversity index was found, when compared to other thermal conditions. The highest mean values of the index at the temperature of 4°C were noted in the last week of investigations at the 0, 8 and 16 h photoperiods, and in the 4th week at the 24 h photoperiod. However, at 16°C and 25°C, the opposite was true: the highest index values were found in the first week at the 0, 8 and 16 h photoperiods, but the highest values were found in the 5th week (at 16^o^C) and 2nd week (at 25°C) for the 24 h photoperiod.

**2.3. Temporal changes in percentage contributions of phytoplankton groups to the total mean phytoplankton abundance in particular experimental treatments and in the field (vernal pool in winter, in the initial stage of phytoplankton succession)**

The share of diatoms and euglenoids in the first two weeks of the investigation was always the highest; in the following weeks, they were replaced by chlorophytes and/or cryptophytes, regardless of photoperiod and temperature, Figs 1A-1C in S2.3, reflecting our field observations from the vernal pool (Fig. 2 in S2.3 compared with data in S3). The share of chlorophytes increased over time at 4°C and at all photoperiods until the 3rd or 4th week, before decreasing. At 16°C, the share of chlorophytes increased over time until the 4th week and then decreased for the 0, 16 and 24 h photoperiods. At 25°C, the share of chlorophytes increased at the 0 and 8 h photoperiods throughout the research period, while at the 16 and 24 h photoperiods, this decreased in the last week.

The greatest share of chlorophytes (above 80% of the total phytoplankton abundance) was always noted at the 8 h photoperiod, especially at 16°C (almost 100%). The share of cryptophytes was the highest at the 16 and 24 h photoperiods, especially at 4°C and 25°C, but their abundance varied over time.

Fig. 1A-1C in S2.3. Temporal changes in percentage contributions of phytoplankton groups to the total mean phytoplankton abundance in particular experimental treatments (A – temp. 4°C, B – temp. 16°C, C – temp. 25°C); 1-5: sampling weeks. The graphs were created using the data in S1.


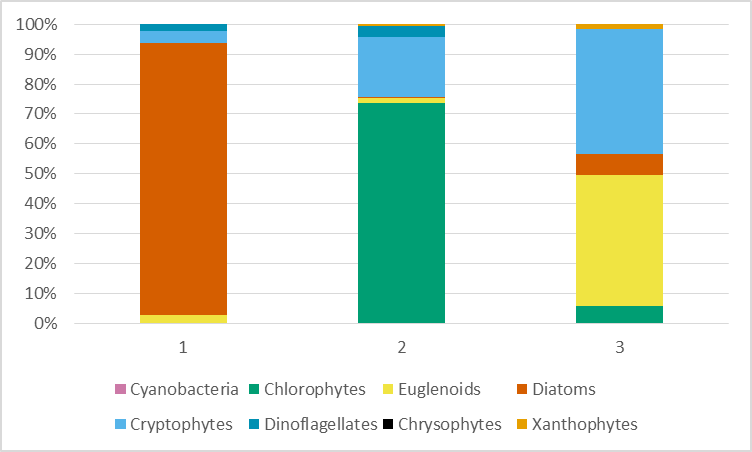


Fig. 2 in S2.3. Temporal changes in percentage contributions of phytoplankton groups to the total mean phytoplankton abundance in the vernal pool in winter; 1-3: sampling days (at two-week intervals). The graph was created using the data in S3.

**2.4. Dominant taxa and their abbreviations on the CCA diagram**

**Cyanoprokaryota/cyanobacteria**: Chrooc1 – *Chroococcus* sp. 1; Hyella - *Hyella* sp.; **Chlorophyta/chlorophytes**: Chla1 – *Chlamydomonas* sp. 1; Chla2 – *Chlamydomonas* sp. 2; Chlapas – *Chamydomonas passiva* Skuja; Chlorac – *Chlorogonium elongatum* var. *aculeatum* (Pascher) L. Péterfi; Chlorel – *Chlorogonium elongatum* (P. A. Dangeard) Francé; Chlorop1 – filamentous chlorophyte 1; Chlorop2 – filamentous chlorophyte 2; Clostac – *Closterium acerosum* Ehrenberg ex Ralfs; Haemat – *Haematococcus pluvialis* Flotow; Mongr - *Monoraphidium griffithii* (Berkeley) Komárková-Legnerová; Oedogon - *Oedogonium* sp.; Phaclen – *Phacotus lenticularis* (Ehrenberg) Diesing; Plansph - *Planctococcus sphaerocystiformis* Korshikov; Pseudla – *Pseudosphaerocystis lacustris* (Lemmermann) Nováková; Schrose – *Schroederia setigera* (Schröder) Lemmermann; Sphagel - *Sphaerellopsis gelatinosa* Korshikov (Gerloff); Spirog – *Spirogyra* sp.; Tetrdim – *Tetradesmus dimorphus* (Turpin) M. J. Wynne; Tetrage – *Tetraspora gelatinosa* (Vaucher) Desvaux; Ulothrix – *Ulothrix* sp.; Uroncon – *Uronema confervicola* Lagerheim; Uronint – *Uronema intermedium* Bourrelly; **Bacillariophyceae/diatoms**: Eunbil - *Eunotia bilunaris* (Ehrenberg) Schaarschmidt; Hantzam – *Hantzschia amphioxys* (Ehrenberg) Grunow in Cleve & Grunow; Nav1 – *Navicula* sp. 1; Nav2 – *Navicula* sp. 2; Navmin – *Navicula minima* Grunow in Van Heurck; Nitzhun – *Nitzschia hungarica* Grunow; Nitzpal – *Nitzschia palea* (Kützing) W. Smith; Stauran – *Stauroneis anceps* f. *gracilis* (Ehrenberg) F. Hustedt; **Cryptophta/cryptophytes**: Chromin – *Chroomonas minuta* (Skuja) Bourrelly; Crypter – *Cryptomonas erosa* Ehrenberg; Cryptma – *Cryptomonas marssonii* Skuja; Cryptov – *Cryptomonas ovata* Ehrenberg; Cryppha – *Cryptomonas phaseolus* Skuja; Rhodmin – *Rhodomonas minuta* Skuja; Rhodten – *Rhodomonas tenuis* Skuja; **Euglenophyta/euglenoids**: Euglen – *Euglena* sp.; Trachv – *Trachelomonas volvocinopsis* Svirenko.
